# Identification of Rab18 as an Essential Host Factor for BK Polyomavirus Infection Using a Whole-Genome RNA Interference Screen

**DOI:** 10.1128/mSphereDirect.00291-17

**Published:** 2017-07-26

**Authors:** Linbo Zhao, Michael J. Imperiale

**Affiliations:** a Department of Microbiology and Immunology, University of Michigan, Ann Arbor, Michigan, USA; b Comprehensive Cancer Center, University of Michigan, Ann Arbor, Michigan, USA; Boston University School of Medicine; Yale School of Medicine; International Centre for Genetic Engineering and Biotechnology, ICGEB

**Keywords:** BKPyV, NRZ complex, Rab18, siRNA screen, syntaxin 18, virus trafficking

## Abstract

BK polyomavirus (BKPyV) is a human pathogen first isolated in 1971. BKPyV infection is ubiquitous in the human population, with over 80% of adults worldwide being seropositive for BKPyV. BKPyV infection is usually asymptomatic; however, BKPyV reactivation in immunosuppressed transplant patients causes two diseases, polyomavirus-associated nephropathy and hemorrhagic cystitis. To establish a successful infection in host cells, BKPyV must travel in retrograde transport vesicles to reach the nucleus. To make this happen, BKPyV requires the cooperation of host cell proteins. To further identify host factors associated with BKPyV entry and intracellular trafficking, we performed a whole-genome small interfering RNA screen on BKPyV infection of primary human renal proximal tubule epithelial cells. The results revealed the importance of Ras-related protein Rab18 and syntaxin 18 for BKPyV infection. Our subsequent experiments implicated additional factors that interact with this pathway and suggest a more detailed model of the intracellular trafficking process, indicating that BKPyV reaches the endoplasmic reticulum (ER) lumen through a retrograde transport pathway between the late endosome and the ER.

**IMPORTANCE** Polyomaviruses bind to a group of specific gangliosides on the plasma membrane of the cell prior to being endocytosed. They then follow a retrograde trafficking pathway to reach the endoplasmic reticulum (ER). The viruses begin to disassemble in the ER and then exit the ER and move to the nucleus. However, the details of intracellular trafficking between the endosome and the ER are largely unknown. By implementing a whole human genome small interfering RNA screen, we identified Rab18, syntaxin 18, and the NRZ complex as key components in endosome-ER trafficking of the human polyomavirus BKPyV. These results serve to further elucidate the route BKPyV takes from outside the cell to its site of replication in the nucleus.

## Abstract (precis)

Polyomaviruses bind to a group of specific gangliosides on the plasma membrane of the cell prior to being endocytosed. They then follow a retrograde trafficking pathway to reach the endoplasmic reticulum (ER). The viruses begin to disassemble in the ER and then exit the ER and move to the nucleus. However, the details of intracellular trafficking between the endosome and the ER are largely unknown. By implementing a whole human genome small interfering RNA screen, we identified Rab18, syntaxin 18, and the NRZ complex as key components in endosome-ER trafficking of the human polyomavirus BKPyV. These results serve to further elucidate the route BKPyV takes from outside the cell to its site of replication in the nucleus.

## INTRODUCTION

BK polyomavirus (BKPyV) is a small icosahedral DNA virus measuring approximately 45 nm in diameter that was first isolated in 1971 (1). Subsequent serology surveys have revealed that up to 80% of the world’s population has been infected with BKPyV (2) and most of the initial infections occur in early childhood (3). After initial exposure, BKPyV establishes an asymptomatic infection in the urinary tract with periodic shedding into the urine (4). BKPyV reactivation when the immune system is compromised in transplant patients, however, causes two diseases, polyomavirus-associated nephropathy (PVAN) and hemorrhagic cystitis (HC) (5). PVAN is one of the leading causes of graft failure after kidney transplantation (6). Also, BKPyV can be detected in the urine of 90% of allogeneic hematopoietic cell transplant patients who suffer from HC, according to a recent study (7). Even though BKPyV was initially isolated more than 45 years ago, the choices for clinical management of BKPyV reactivation are limited. The first-line treatment for PVAN is lowering the dosage of immunosuppressants, which inevitably increases the risk of acute rejection and graft failure (6). Other options for treating BKPyV reactivation are treatment with cidofovir, leflunomide, and fluoroquinolones; however, the benefit of using these drugs in addition to reducing immunosuppressants has been questioned and has not been investigated carefully in randomized studies (6, 8). Because of the lack of specific antiviral medicines, management of BKPyV reactivation remains a challenge.

BKPyV does not encode any polymerases. As a result, BKPyV exclusively relies on the host DNA replication machinery for its genome replication. To establish a successful infection, BKPyV must deliver its DNA genome into the nucleus to access the host DNA replication machinery, which entails crossing the plasma membrane, the endoplasmic reticulum (ER) membrane, and the nuclear envelope (9). Also, BKPyV needs to navigate a crowded cytoplasm to reach the ER. Without any cooperation from host factors, this process would seem difficult, if not impossible.

Few host factors have previously been identified that participate in viral entry and intracellular trafficking. To initiate infection, BKPyV binds to ganglioside GD1b or GT1b on the host cell membrane (10). After binding to the cell membrane, BKPyV had been thought to enter host cells via a caveolin-mediated pathway (11); however, subsequent experiments showed that caveolin is dispensable for infection of human renal proximal tubule cells (12). After endocytosis, BKPyV enters the endosome in the same way as other polyomaviruses (13–16). Acidification and maturation of the endosome are essential for BKPyV infection (17) and activate a sorting machinery that involves the Rab5, Rab7, Rab9, and Rab11 proteins (14, 15, 18). After sorting through the late endosome, vesicles that contain BKPyV traffic along microtubules and reach the ER at 8 to 12 h postinfection (17, 19). ER lumen proteins are essential for polyomavirus disassembly and egress from the ER (9, 17, 20–22). Briefly, protein disulfide isomerases induce capsid conformational changes and minor capsid protein exposure (17, 23–28). These exposed minor proteins insert themselves into the ER membrane (29, 30), and partially disassembled polyomaviruses penetrate the ER membrane and enter the cytosol through the ER-associated degradation pathway (9, 20, 21, 31, 32). In the cytosol, the nuclear localization signal of the minor capsid proteins guides polyomaviruses into the nucleus via the importin α/β pathway (33–35). In addition to this productive model of entry, a significant portion of internalized polyomavirus enters a nonproductive pathway (36). Distinguishing the critical events that BKPyV uses to establish successful infection from other pathways that BKPyV uses remains a challenge.

Numerous assays have been developed for the purpose of identifying host factors associated with viral infections, including genome-wide small interfering RNA (siRNA) screening. By silencing every single human gene with an siRNA pool and then assessing the effects of the knockdown on infection, it is feasible to dissect the functions of individual host proteins during the viral life cycle. siRNA screening has been extensively applied to research on viruses such as HIV (37), West Nile virus (38), human papillomavirus (HPV) (39), vesicular stomatitis virus (40), and another polyomavirus, simian virus 40 (SV40) (41). However, no genome-wide siRNA screen has been reported for BKPyV.

Using a whole-genome siRNA screen, we have identified a series of potential host factors that are involved in BKPyV infection. DNAJ B14, which has previously been implicated in BKPyV entry, is our top hit, and DNAJ B12 is also among our top 100 hits (21, 41). Most of the other hits we have identified have not been previously reported, however, and many of them are involved in vesicular transport. In this report, we present data showing that two of our primary hits, Rab18 and syntaxin 18, as well as two members of the NRZ complex (RAD50 interactor 1 and ZW10 kinetochore protein), which have previously been shown to interact with Rab18 and syntaxin 18, are essential host factors for BKPyV infection.

## RESULTS

### A high-throughput siRNA screen for BKPyV based on cell cycle analysis.

To identify host factors involved in viral entry and intracellular trafficking, we developed and implemented a high-throughput whole-genome siRNA screen for BKPyV infection in its natural host cells, primary human renal proximal tubule epithelial (RPTE) cells. In this screen, BKPyV infection was challenged with the Dharmacon siGENOME SMARTpool siRNA library, which includes more than 18,000 human siRNA pools. Each pool contains four unique siRNAs that target the same host gene. The screen was performed in triplicate. Every screen plate included the siGENOME nontargeting control (NTC) as a negative control and a synthetic siRNA that corresponds to the natural BKPyV 5p microRNA (miRNA) targeting the large tumor antigen (TAg) mRNA (siTAg) (42) as a positive control.

Our lab previously showed that BKPyV induces G_2_/M arrest to take full advantage of the host DNA replication machinery (43); this provided us with a cost-efficient approach to evaluate viral infection in siRNA-transfected cells. By applying a Hoechst DNA stain, cells in G_2_/M phase are distinguishable from the rest of the cells on the basis of the difference in DNA content in each nucleus. The percentage of G_2_/M-arrested cells (G_2_%) dramatically increases after BKPyV infection, and we adopted it as the readout for the screening assay.

For the primary screen, RPTE cells were transfected with the siRNA library and cultured for 48 h to allow depletion of the targeted proteins. Next, cells were infected with BKPyV and cultured for an additional 2 days. The cells were fixed and stained with Hoechst, and the numbers of nuclei in the different stages of the cell cycle were quantified, recorded, and uploaded to the MScreen web tool developed by the Center for Chemical Genomics (University of Michigan) (44). G_2_% values were calculated with the MScreen web tool. To proceed with the data analysis, we scaled data from 0 to 100, with 100 representing the effect of our positive control and 0 representing the effect of our negative control. The effect of each siRNA pool was then calculated using this index scale. A larger index value represents a stronger inhibitory effect on BKPyV infection when an siRNA pool is introduced into RPTE cells, which suggests that the targeted host factor is more likely to be required for BKPyV infection.

**FIG 1:**
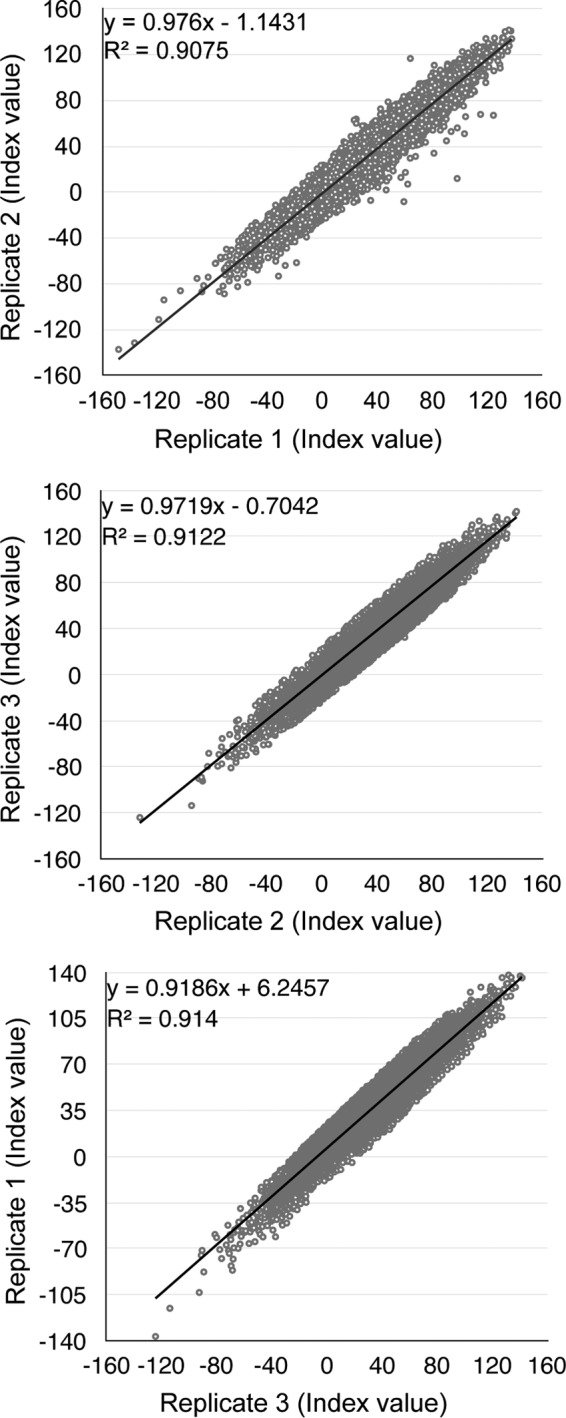
Visualization of whole-genome RNAi screen results. The effects of each siRNA pool are normalized to the NTC siRNA (set as 0) and siTAg (set as 100). Index values from the replicates are illustrated in pairwise scatterplots.

The Z factor is a statistical parameter widely used for screen assay evaluation. A minimal Z factor of 0.5 is required for a reliable siRNA screen (45). The overall Z factor achieved in our whole primary screen was 0.6, and the R^2^ values between replicates were approximately 0.9. We compared the data from the three replicates in a pairwise manner, as illustrated in the scatterplots in Fig. 1. Seventy percent of the siRNA pools in the siGENOME siRNA library inhibited BKPyV infection to some degree compared to the nontargeting siRNA control. A similar distribution of inhibitory effects was also observed in an siRNA screen using the siGENOME library to assess cellular genes involved in HPV infection (39). For the results of the primary screen, see Table S1 in the supplemental material.

10.1128/mSphereDirect.00291-17.1 TABLE S1 Primary screening results. The primary screen was performed in triplicate. The effects of each siRNA pool were normalized to the nontargeting siRNA control (set as 0) and siTAg (set as 100). After normalization, the average value and standard deviation were calculated for each siRNA pool. Download TABLE S1, XLSX file, 1.1 MB. Copyright © 2017 Zhao and Imperiale. 2017 Zhao and Imperiale This content is distributed under the terms of the Creative Commons Attribution 4.0 International license.

We parsed the candidate genes into functional categories and pathways with the Database for Annotation, Visualization, and Integrated Discovery (DAVID) enrichment analysis tool (46, 47). DAVID analysis showed that translation-related genes were the most enriched group of genes that are required for viral infection among the candidate genes (Table S2). Considering that BKPyV exclusively relies on the host protein synthesis machinery, this result is consistent with our expectation. Other than translation-related genes, several clusters of genes that are required for viral infection were also significantly enriched, especially those for budding and coat proteins associated with vesicular transport. On the basis of the enrichment analysis results, we manually selected 147 siRNA pools for secondary validation.

10.1128/mSphereDirect.00291-17.2 TABLE S2 DAVID analysis results. The top 800 hits from the primary screen were analyzed with the DAVID enrichment analysis tool. The most-enriched categories are listed. Download TABLE S2, DOCX file, 0.1 MB. Copyright © 2017 Zhao and Imperiale. 2017 Zhao and Imperiale This content is distributed under the terms of the Creative Commons Attribution 4.0 International license.

G_2_% is an indirect readout of BKPyV infection. Knocking down cell cycle regulatory proteins may also affect G_2_/M arrest independently of viral infection; thus, false-positive or false-negative candidates could be introduced into the results during the primary screen. To eliminate these false candidates, we next applied a direct readout, immunofluorescent staining for the early viral protein TAg, to validate these 147 genes. RPTE cells were transfected and infected in 96-well plates with a subset of the siGENOME library that contains the selected 147 siRNA pools. Infected cells were fixed at 48 h postinfection and probed for TAg with a primary antibody and a fluorescein isothiocyanate (FITC)-labeled secondary antibody. After images were acquired, the integrated TAg fluorescent intensity per nucleus was recorded and the inhibition index was calculated as in the primary screen (Table S3).

10.1128/mSphereDirect.00291-17.3 TABLE S3 Validation of the primary hits. The effects of 147 manually selected siRNA pools on BKPyV infection of RPTE cells were examined. The validation screen was performed in triplicate. Infected cells were fixed at 48 h postinfection and stained for TAg with a primary antibody and an FITC-labeled secondary antibody. The integrated TAg fluorescent intensity per nucleus was recorded, and the inhibition index was calculated as in the primary screen. After normalization, the average value and standard deviation were calculated for each siRNA pool. Download TABLE S3, XLSX file, 0.02 MB. Copyright © 2017 Zhao and Imperiale. 2017 Zhao and Imperiale This content is distributed under the terms of the Creative Commons Attribution 4.0 International license.

After the secondary screen, DNAJ B14, which has previously been implicated in BKPyV entry, was our top hit and DNAJ B12 and DNAJC3, which are also involved in polyomavirus trafficking, were also among our top 10 hits (21, 41), which indicates the overall robustness of our screen. Rab18 was one of our top validated hits other than these DNAJ proteins. Moreover, syntaxin 18, a protein that has previously been demonstrated to interact with Rab18, also passed our validation.

### The Rab18/NRZ/syntaxin 18 complex is required for BKPyV infection.

The Rab protein family is a group of small GTPases that regulate membrane trafficking and that have been implicated in polyomavirus intracellular trafficking (14, 18, 48). Rab18, however, had not previously been associated with polyomavirus infection. The Rab18 interaction network has been thoroughly investigated (49), and more than 40 proteins have been identified as interacting with Rab18. Among these proteins, syntaxin 18 was also one of our top hits in the screen.

**FIG 2:**
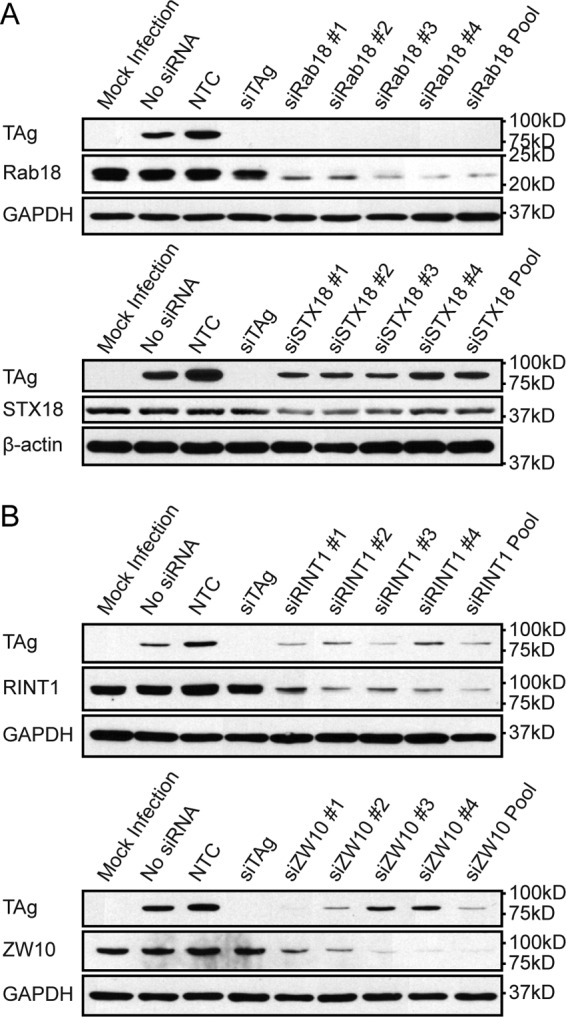
Rab18, syntaxin 18 (STX18), and the NRZ complex are required for BKPyV infection. RPTE cells were transfected with the siRNAs indicated and then infected with BKPyV. Viral infection (TAg), GAPDH or β-actin expression levels, and knockdown efficiency were examined by Western blotting.

To determine whether Rab18 and syntaxin 18 are required during BKPyV infection, RPTE cells in 12-well plates were transfected with pooled or individual siRNAs targeting these proteins. Cells transfected with the NTC siRNA pool and siTAg served as negative and positive controls, respectively. After transfection, cells were cultured for 2 days to allow for depletion of the targeted proteins and then infected with BKPyV at a multiplicity of infection (MOI) of 1 infectious unit (IU)/cell. Cells were lysed at 48 h postinfection, and expression of the targeted proteins, TAg, and β-actin or glyceraldehyde-3-phosphate dehydrogenase (GAPDH) was assessed by Western blotting (Fig. 2A). The choice of β-actin or GAPDH was based on avoidance of comigration of the loading control with the protein of interest. Knockdown of Rab18 and syntaxin 18 reduced TAg expression, indicating that both proteins are required for efficient infection. Knockdown of Rab18 entirely blocked BKPyV infection. While there was only partial knockdown of syntaxin 18 with both the pooled and individual siRNAs, TAg expression decreased correspondingly. Because syntaxin 18 is a SNARE protein (50), this suggests that BKPyV enters the ER lumen via a vesicle fusion step.

In addition to syntaxin 18, Rab18 interacts with ZW10 kinetochore protein (ZW10) and RAD50 interactor 1 (RINT1), which, together with neuroblastoma amplified gene (NAG), are members of the NRZ complex (51). Upon activation by GTP, Rab18 interacts with the ZW10 kinetochore protein from the NRZ complex, thereby forming a Rab18/NRZ/syntaxin 18 complex at the ER (49). In this complex, syntaxin 18 functions as a t-SNARE on the ER membrane where the NRZ components work as a tether to assist with retrograde vesicle docking (51, 52). NRZ/syntaxin 18 cooperate in capturing Rab18-labeled vesicles and initiating the fusion process on the ER membrane. Although none of the NRZ complex proteins were identified in our primary screen, we hypothesized that they are required for BKPyV infection because of their ability to interact with both Rab18 and syntaxin 18. To test whether NRZ components are involved in BKPyV infection, we knocked down RINT1 and ZW10 and then infected cells. We could not test NAG because of the lack of a useful antibody to measure its expression. The Western blot results indicate that disruption of the NRZ complex by knocking down these two components interferes with infection (Fig. 2B).

### Rab18 colocalizes with the viral capsid during BKPyV intracellular trafficking.

**FIG 3:**
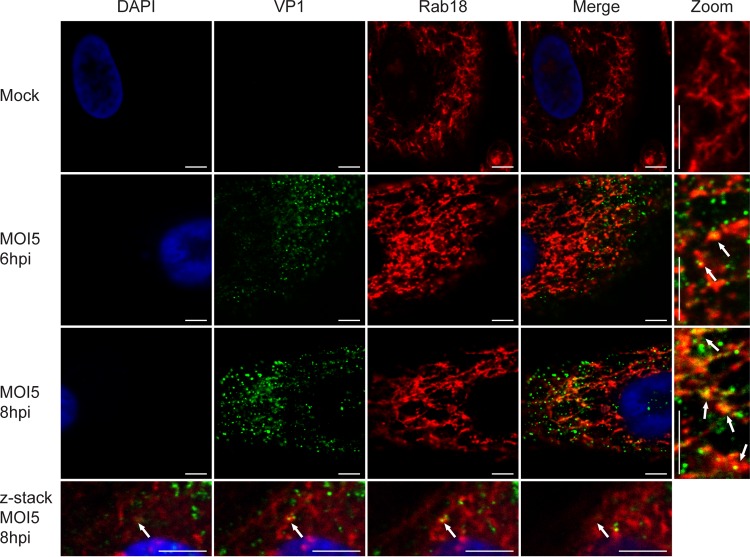
Colocalization of Rab18 and BKPyV capsid protein VP1. RPTE cells were fixed at 6 and 8 h postinfection and stained for VP1 (green), Rab18 (red), and DAPI (blue). Images were taken by confocal microscopy. Sequential z-stack images (0.25-μm increments) from the bottom of the cell to the top (left to right) are shown. Arrows point to VP1-Rab18 colocalization sites. Bars represent 5 µm.

We next asked whether Rab18 is associated with BKPyV-containing vesicles, since Rab18 targets vesicles to the ER (53, 54). To test this, we fixed RPTE cells at 6 and 8 h postinfection and stained them for BKPyV major capsid protein VP1 and Rab18. Images of stained RPTE cells were acquired with a confocal microscope. The confocal images show that Rab18 and VP1 are partially colocalized at 6 and 8 h postinfection (Fig. 3). This is consistent with the asynchronous trafficking of BKPyV from endosomes after entry (17). These results support our hypothesis that BKPyV travels in Rab18-positive vesicles after being sorted through endosomes.

### BKPyV traffics differently after Rab18, syntaxin 18, or NRZ knockdown.

**FIG 4:**
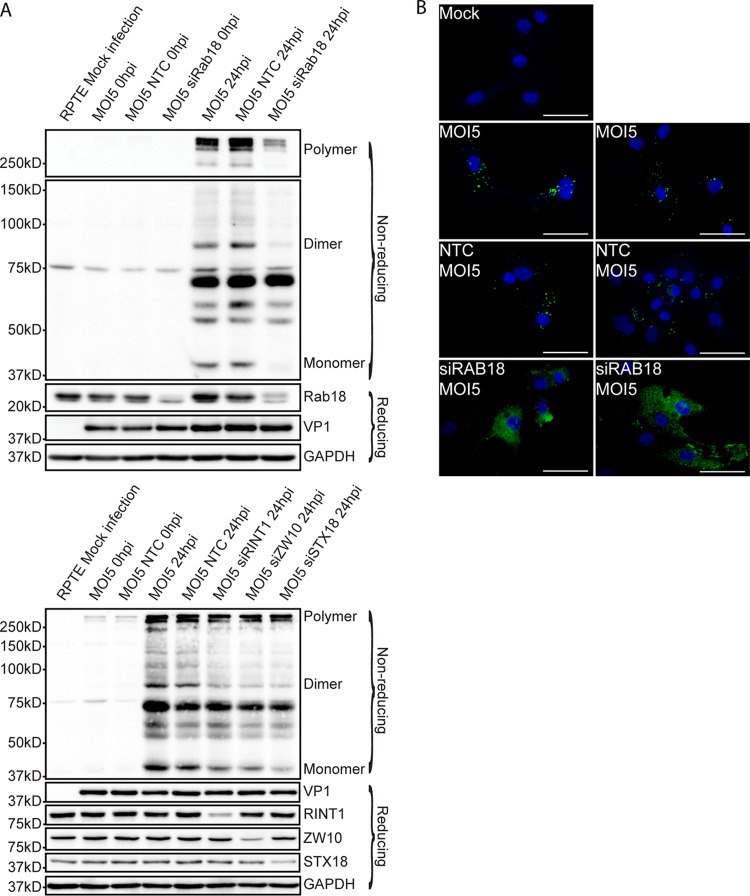
Effects of Rab18 knockdown on BKPyV intracellular trafficking. (A) Rab18, syntaxin 18 (STX18), and NRZ knockdown prevents ER delivery and BKV capsid rearrangement. RPTE cells were transfected with the siRNAs indicated. After 48 h, they were infected with BKPyV and lysed immediately after adsorption or at 24 h postinfection under reducing or nonreducing conditions. BKPyV capsid protein VP1, GAPDH, Rab18, STX18, RINT1, and ZW10 levels were examined by Western blotting. (B) Alteration of BKPyV intracellular trafficking. RPTE cells were transfected with NTC or siRAB18. BKPyV particles were visualized by immunofluorescent staining for VP1 (green) at 24 h postinfection. Bars represent 100 µm.

To establish a successful infection, BKPyV must reach the ER lumen, where disassembly occurs (17). After internalization into host cells, conformational changes of the capsid are neither required nor observed before polyomaviruses enter the ER lumen (27, 28). When polyomavirus particles reach the ER lumen, luminal enzymes disrupt the disulfide bonds between VP1 monomers, thereby initiating disassembly of the viral capsid (26). This process in the ER can be visualized by assaying for VP1 monomer, dimer, and oligomer bands after separation of proteins under nonreducing conditions by SDS-PAGE (17, 28). To confirm that BKPyV requires Rab18 to reach the ER lumen and initiate disassembly, we examined BKPyV disassembly after Rab18 knockdown. RPTE cells were transfected with NTC or Rab18 siRNA for 48 h and infected with BKPyV at an MOI of 5 IU/cell. Protein samples were processed and assayed at 24 h postinfection by Western blotting of reducing and nonreducing gels (17, 28). At 0 h postinfection, most of the BKPyV particles were intact and too large to enter the nonreducing SDS-PAGE gel, and no VP1 monomers (42 kDa) or dimers were visible. At 24 h postinfection in cells in which Rab18 was present, BKPyV began to disassemble and VP1 monomers, dimers, and oligomers could be detected. However, the nonreducing gel suggests that the BKPyV particle could not disassemble efficiently without Rab18 (Fig. 4A, top). The reducing gel shows that equal amounts of VP1 are detectable in the presence or absence of Rab18, indicating that knocking down Rab18 did not prevent BKPyV from attaching to or entering RPTE cells. We also assessed the effect of knocking down RINT1, ZW10, and syntaxin 18 on BKPyV disassembly (Fig. 4A, bottom). In each case, smaller amounts of VP1 monomers and dimers were detected in the nonreducing gel, suggesting that BKPyV cannot disassemble efficiently without the help of the NRZ complex.

Previous studies showed that mouse polyomavirus (MPyV) could enter cells that do not produce proper ganglioside receptors; afterward, these MPyV particles are trapped in a dead-end pathway and cannot establish a successful infection. This indicates that polyomaviruses enter both productive and nonproductive pathways inside host cells. These nonproductive pathways result in a diffuse distribution of viral particles, as detected by immunofluorescent staining (36). In the presence of proper ganglioside receptors, polyomaviruses leave the cell periphery, move along microtubules, and reach the perinuclear area (17, 19, 55, 56). Ultimately, the productive pathway leads to the ER (17, 28, 57). Since we saw that BKPyV could not reach the ER lumen without Rab18 while the entry of BKPyV was not affected (Fig. 4A), we wished to address if BKPyV traffics in a different pattern under Rab18 knockdown. To test this, Rab18 was depleted as previously described and RPTE cells were infected with BKPyV at an MOI of 5 IU/cell. Cells were fixed and stained for VP1 at 24 h postinfection (Fig. 4B). In the presence of Rab18, BKPyV forms bright foci in the perinuclear area, as previously observed (9). However, Rab18 knockdown caused dispersion of the bright perinuclear VP1 foci and the staining of VP1 appeared to be diffuse and spread throughout the cytoplasm (Fig. 4B). Thus, without Rab18, BKPyV traffics in a pattern that is suggestive of a nonproductive pathway (36).

### BKPyV is enriched in the late endosome without Rab18.

**FIG 5:**
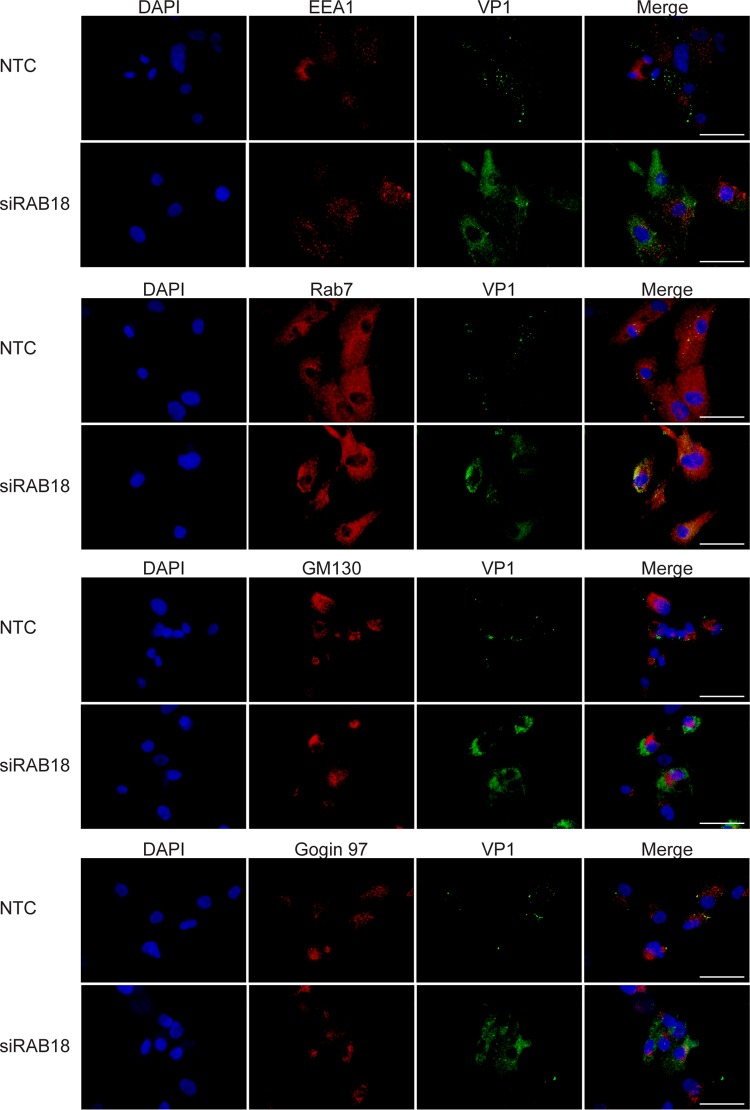
Colocalization of BKPyV and organelle markers. RPTE cells were transfected with NTC or siRAB18 and then stained for VP1 (green) and the organelle markers indicated above the rows of panels (red) at 24 h postinfection. Images were taken with an inverted fluorescence microscope. Bars represent 100 µm.

**FIG 6:**
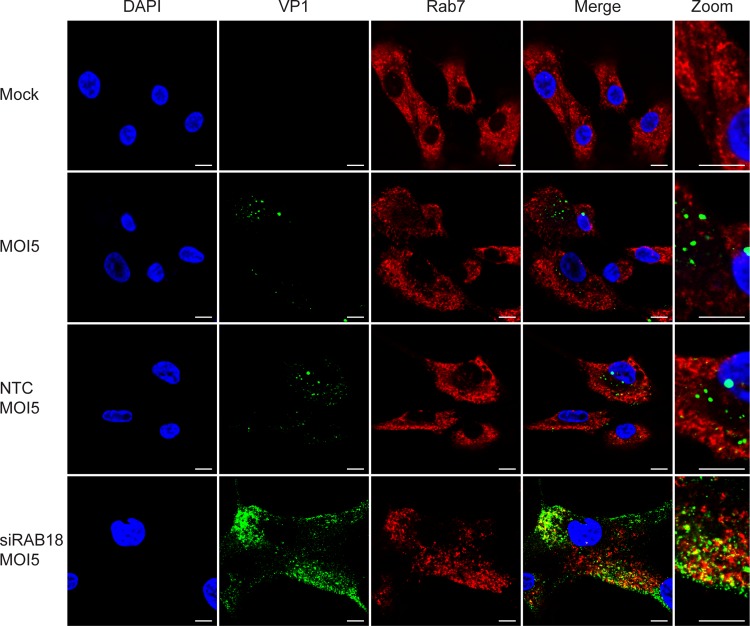
Colocalization of VP1 and Rab7. RPTE cells were transfected with NTC or siRAB18 and then stained for VP1 (green) and Rab7 (red) at 24 h postinfection. Images were taken with a confocal microscope. Bars represent 10 µm.

Our previous study demonstrated that acidification of the endosome is essential for BKPyV infection (17) and MPyV colocalizes with the late endosome marker Rab7 after entry (18). Furthermore, BKPyV cannot traffic efficiently to the ER and initiate capsid disassembly without abundant Rab18 and instead appears to be trapped in an unknown compartment(s) diffusely located in the cytoplasm (Fig. 4). To identify this compartment, we knocked down Rab18 in RPTE cells, fixed the cells at 24 h postinfection, and probed for various organelle markers (Fig. 5). We found that the diffuse viral particles do not colocalize with the early endosome marker EEA1, the *cis*-Golgi marker GM130 (58), or the *trans*-Golgi marker Golgin 97 (59). On the other hand, we found that the VP1-rich area overlaps Rab7. To further confirm this colocalization, images of VP1 and Rab7 were taken by confocal microscopy (Fig. 6). We found that the dispersed VP1 partially colocalizes with Rab7 when Rab18 is knocked down, suggesting that some viral particles are trapped in the late endosome in the absence of Rab18. There is also some VP1 that does not colocalize with Rab7. This may be a result of asynchronous trafficking of BKPyV, and additional intermediate and dead-end compartments could also exist during the retrograde trafficking step between the endosome and the ER. Our results suggest that BKPyV reaches the ER lumen through a Rab18-mediated retrograde transport pathway between the late endosome and the ER.

## DISCUSSION

BKPyV was initially isolated more than 45 years ago (1); however, our understanding of the early events in the BKPyV life cycle contains gaps. Polyomaviruses appear to take advantage of multiple endocytic pathways to enter the host cell. However, some of the pathways that BKPyV uses are nonproductive (36), which suggests that identifying host factors solely by morphological observations, especially protein colocalization evidence, may be deceiving. SV40, BKPyV, MPyV, and cholera toxin have been observed to enter the cell via a caveolin-mediated pathway (11, 60–64). However, subsequent experiments have demonstrated that caveolin is dispensable (12, 14, 65–68). Because of the limitations of the available biological tools, research on the earliest stages of the polyomavirus life cycle has progressed relatively slowly.

Whole-genome siRNA screening provides an unbiased tool with which to investigate some of the remaining questions. However, one of the biggest challenges of developing an siRNA screen assay is finding a readout that is sensitive, cost efficient, and suitable for high-throughput automation. One of the most common readouts for a viral siRNA screen is cell viability. However, BKPyV, along with many viruses, does not lyse host cells within a reasonable period compared to the duration of the RNA interference (RNAi) effect in cells. This behavior of BKPyV makes cell viability measurements impractical. Another common strategy to evaluate viral infection is incorporation of reporter genes into the viral genome. Because BKPyV is highly sensitive to genomic modification, several approaches attempting to integrate reporter genes into the BKPyV genome have failed (data not shown). Therefore, construction of modified viral particles containing a reporter gene was not practical for us. Finally, immunofluorescent staining for viral protein expression is neither cost nor time efficient for processing hundreds of plates. For all of these reasons, we needed a different readout for evaluation of viral infection.

The percentage of cells in G_2_/M phase provided us a cost-efficient assay for the primary screen. Our primary screen results indicated that knocking down 70% of the human genes in RPTE cells inhibited BKPyV infection to some degree compared to the NTC. A similar data distribution was also observed in an siRNA screen for HPV infection with the same siRNA library (39). We found that the NTC siRNA reproducibly slightly increased BKPyV infection compared to the no-siRNA control. This could be because of interference of exogenous siRNA with the host intrinsic miRNA processing and silencing mechanism. BKPyV encodes a miRNA that downregulates TAg mRNA levels (42, 69). If the introduction of exogenous siRNA affects the miRNA processing and silencing mechanism, TAg expression would increase because of lower levels of mature BKPyV miRNA.

The increased BKPyV infection induced by the nontargeting siRNA control could also be an off-target effect of RNAi. While validating our primary screening results, we found that many of our primary hits were not reproducible when tested with individual siRNAs instead of siRNA pools. It appears that off-target effects of RNAi are inevitable for whole-genome siRNA screens, and an “ideal nontargeting” siRNA pool does not exist (70). The most likely cause of the off-target effect is the passenger strand of the siRNA. Each siRNA carries a complementary (passenger) strand to enhance the stability of siRNA molecules. This passenger strand can also be integrated into the RNA-induced silencing complex (RISC), which possibly increases off-target effects. Moreover, even if only the guide strand enters the RISC complex, RISC prefers the second to the eighth nucleotides of that strand, called the seed region, to recognize targeted mRNAs (70–74). Considering this characteristic of RNAi, each seven-nucleotide seed region can target more than one gene. Off-target effects can therefore increase the expense of validation and lower the efficiency of screening.

After validating some of the top hits, as well as other proteins that are known to be involved in the same vesicular trafficking pathway, Rab18, syntaxin 18, ZW10, and RINT1 were confirmed as essential host factors for BKPyV infection. Rab18 has been associated with lipid droplet homeostasis and vesicular transport between the Golgi apparatus and the ER (53, 54). The majority of Rab18 usually localizes to the membrane of the ER and the *cis*-Golgi apparatus (53). Also, Rab18 is recruited to the surface of lipid droplets, the endosome, and the lysosome (75, 76). Rab18 has been shown to interact with syntaxin 18, ZW10, and RINT1 by affinity chromatography followed by mass spectrometry (49). On the basis of previous studies on yeast proteins, ZW10 and RINT1 interact with each other via their N termini and interact with the NAG protein to form the NRZ complex. The t-SNARE protein syntaxin 18 on the ER membrane indirectly attaches to the NRZ complex (51, 52, 77). The overall structure of the NRZ complex is a protrusion about 20 nm in length from the surface of the ER membrane (78). GTP-activated Rab18 on the surface of vesicles interacts with ZW10, thereby tethering vesicles to the ER membrane. Syntaxin 18 is a SNARE protein and mediates vesicle fusion to the ER (50). After the NRZ complex captures vesicles, syntaxin 18 mediates vesicle fusion with the ER membrane.

**FIG 7:**
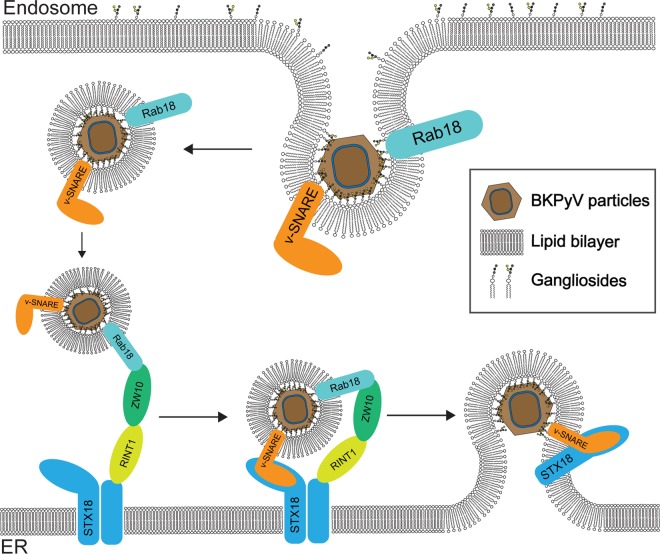
Model of BKPyV vesicular trafficking. BKPyV enters a vesicle from the membrane of the endosome. GTP-bound Rab18 then interacts with ZW10 of the NRZ tethering complex. Syntaxin 18 on the ER membrane interacts with v-SNARE, and syntaxin 18 and v-SNARE mediate vesicle fusion.

We propose a model of BKPyV entry and intracellular trafficking to the ER that is based on previous findings and our present results (Fig. 7). To initiate infection, BKPyV first binds to its ganglioside receptors GT1b and GD1b (10). After initial attachment, BKPyV is believed to form deep invaginations of the host plasma membrane in the same manner as SV40 (79). The length of ceramide tails of the ganglioside plays a critical role in the formation of the invagination (79). Next, BKPyV enters host cells via a ganglioside-dependent, caveolin- and clathrin-independent pathway (12, 80). Meanwhile, glycoproteins on the plasma membrane appear to serve as traps for polyomavirus (81); viral particles that bind to glycoproteins will eventually enter nonproductive pathways. Only those particles that bind to the correct ganglioside receptor can enter the productive infection pathway (36). Furthermore, binding to the gangliosides is sufficient to drive the transport of artificial particles to the ER (18). This indicates that binding to the proper ganglioside is both necessary and sufficient for BKPyV to traffic in retrograde transport vesicles to the ER. In addition, conformational changes of the capsid are neither required nor observed before polyomaviruses enter the ER lumen (27, 28). These findings reveal that BKPyV may remain bound to the membrane throughout intracellular trafficking until it reaches the ER lumen. BKPyV enters the endosome in the same manner as other polyomaviruses (13–15). Acidification of the endosome activates a ganglioside-sorting machinery that sorts BKPyV into secondary vesicles along with gangliosides (17, 18, 82). Without Rab18, we found that BKPyV accumulates in the late endosome (marked by Rab7). This suggests that Rab18 mediates late-endosome-to-ER trafficking of BKPyV and that it buds and traffics together with virus-containing vesicles along microtubules (17, 19, 55, 83). After the NRZ complex on the ER membrane captures and tethers vesicles to the ER surface, syntaxin 18 further interacts with a yet-to-be-identified v-SNARE on the vesicles and the syntaxin 18/v-SNARE complex mediates vesicle fusion to the ER membrane. This allows successful entry of BKPyV into the ER lumen. Our finding supports the conclusion that late-endosome-to-ER trafficking plays a critical role in BKPyV infection.

It has been shown that fluorescently labeled gangliosides can spontaneously move in a retrograde direction to the ER (77). BKPyV and the other polyomaviruses are therefore like hitchhikers that take a ride along this ganglioside-mediated retrograde pathway to the ER. Whether additional interactions between the viral capsid and host proteins play a role in BKPyV trafficking to the ER is still unclear. Gangliosides play important roles in both endocytosis and retrograde trafficking. Polyomaviruses and several toxins are proposed to take advantage of lipid-mediated endocytosis and retrograde trafficking pathways (reviewed by Ewers and Helenius [80]). Besides polyomaviruses, some other nonenveloped viruses infect cells in a similar manner; therefore, they may also utilize part of this lipid-mediated retrograde trafficking pathway to establish an infection (84); the retrograde paths of both HPV and BKPyV can be blocked with the retrograde traffic inhibitors retro-1, retro-2, and brefeldin A (17, 39, 85, 86). Norovirus also binds to gangliosides (87, 88) and forms deep invaginations on model membranes (89). Gangliosides have been demonstrated to be important for rotavirus infection (90), and rotavirus also traffics to and is sorted through the endosome (91). However, details of the Rab18-mediated pathway are largely unknown and further efforts are needed to fully reveal the details of this retrograde trafficking pathway.

## MATERIALS AND METHODS

### Cell culture.

Primary RPTE cells purchased from Lonza were maintained in the recommended medium, REGM BulletKit (REGM/REBM, CC-3190; Lonza), at 37°C with 5% CO_2_ in a humidified incubator. Cells recovered from a single frozen vial from Lonza (passage 2) were cultured for three passages. Afterward, cells at passage 5 were passaged one more time and plated for screening or aliquoted and frozen in liquid nitrogen for later experiments. For all experiments other than the primary screen, frozen aliquots (passage 5) were recovered about 1 week before each experiment and cells were then plated for experiments.

### Infection.

BKPyV (Dunlop) was cultured and purified on a cesium chloride linear gradient, and titers were determined as described previously (17, 92). RPTE cells were infected as follows at 2 days after siRNA transfection. Cells were prechilled for 15 min at 4°C. Purified viruses were diluted to 175,000 IU/ml (MOI of 1 IU/cell) or 875,000 IU/ml (MOI of 5 IU/cell) in REBM/REGM. A 400-μl volume of the diluted virus was added to the wells of a 12-well plate and incubated at 4°C for 1 h with shaking every 15 min to distribute the inoculum over the entire well. The plate was transferred to 37°C after the 1-h incubation.

### siRNA and siRNA library.

The whole-genome human siGENOME SMARTpool siRNA library from Dharmacon was acquired and prepared by the Center for Chemical Genomics (University of Michigan). All other siRNAs were also purchased from Dharmacon, i.e., a nontargeting siRNA control (D-001206-14); Rab18 siRNA (LQ-010824-00); STX18 siRNA (LQ-020624-01); ZW10 siRNA (LQ-003948-00); RINT1 siRNA (LQ-004976-01); and siRNA targeting large T antigen (custom synthesized with the sequence 5′ AUCUGAGACUUGGGAAGAGCAU 3′), which corresponds to the natural BKPyV 5p miRNA (42).

### Primary siRNA screening in 384-well plates.

The siRNA library was rehydrated at 500 nM in siRNA buffer (Dharmacon catalog no. B-002000-UB-100) in accordance with the Basic siRNA resuspension protocol from Dharmacon. A 1-µl volume of a 500 nM siRNA suspension was spotted into each well of 384-well PE View plates on a Biomek laboratory automation workstation. RPTE cells were transfected in accordance with the Lipofectamine RNAiMAX (Thermo Fisher Scientific) manual. Briefly, transfection complexes were prepared by adding 9 µl of diluted transfection reagent (0.78% [vol/vol] RNAiMAX reagent in REBM/REGM without antibiotics) to each well of the 384-well plates into which the siRNAs had been spotted. The transfection complexes were incubated at room temperature for 20 min before 1,800 cells suspended in 10 µl of REBM/REGM without antibiotics were added to each well. Transfected cells were cultured at 37°C for 48 h, after which cells were infected as follows. Plates were incubated at 4°C for 15 min; purified BKPyV Dunlop was diluted in cold REGM/REBM; 5 µl containing virus at 1,800,000 IU/ml was dispensed into each well with a Multidrop Combi reagent dispenser (Thermo Fisher Scientific); and the plates were incubated at 4°C for 1 h and then transferred to 37°C for an additional 48 h. Next, cells were fixed with 4% paraformaldehyde (Electron Microscopy Sciences) at room temperature for 20 min, permeabilized with 0.1% Triton X-100 (MilliporeSigma) in phosphate-buffered saline (PBS) for 5 min at room temperature, and stained with 2 µg/ml Hoechst 33342 (Thermo Fisher Scientific, H3570) in PBS for 15 min at room temperature. Cells were washed three times with PBS after each staining step. Images of the wells were taken with an ImageXpress Micro XLS high-throughput microscope and analyzed with MetaXpress High-Content Image Acquisition and Analysis software. The quantified data generated from MetaXpress were uploaded and further analyzed with MScreen, a high-throughput analysis system developed by the Center for Chemical Genomics (University of Michigan) (44). Low-quality data points generated from wells with contamination or significant cell death were discarded.

### Secondary siRNA screening in 96-well plates.

For siRNA transfection in 96-well plates, the primary screening protocol was scaled up by applying three times the volume used in 384-well plates. In addition to the Hoechst stain, cells were also incubated for 1 h each with 5% goat serum, anti-TAg monoclonal antibody (pAb416) at a 1:200 dilution in 5% goat serum (93), and goat anti-mouse IgG-FITC (MilliporeSigma F2012) at a 1:200 dilution in 5% goat serum. Cells were rinsed three times with 1× PBS between steps. Images of the wells were taken with an ImageXpress Micro XLS high-throughput microscope and analyzed for TAg intensity with MetaXpress High-Content Image Acquisition and Analysis software.

### Tertiary validation in 12-well plates.

siRNAs were rehydrated at 1 µM in siRNA buffer (Dharmacon catalog no. B-002000-UB-100). Transfection complexes were prepared by mixing 20 µl of 1 µM siRNA with 380 µl of diluted transfection reagent (0.74% [vol/vol] RNAiMAX reagent in REBM/REGM without antibiotics) in each well of a 12-well plate. The transfection complexes were incubated at room temperature for 20 min before 70,000 cells suspended in 400 µl of REBM/REGM without antibiotics were added to each well. RPTE cells were infected at 2 days posttransfection.

### Preparation of protein lysates.

Cells were lysed at 48 h postinfection with E1A buffer (50 mM HEPES [pH 7], 250 mM NaCl, and 0.1% NP-40 with inhibitors [5 µg/ml phenylmethylsulfonyl fluoride, 5 µg/ml aprotinin, 5 µg/ml leupeptin, 50 mM sodium fluoride, and 0.2 mM sodium orthovanadate] added right before use; MilliporeSigma). For nonreducing gels, cells were rinsed with 1× PBS and 10 mM *N*-ethylmaleimide (NEM; MilliporeSigma catalog no. E3876) and harvested by scraping. Harvested cells were further pelleted with a centrifuge and lysed with a Triton lysis buffer (10 mM Tris [pH 7.6], 10 mM sodium phosphate, 130 mM NaCl, 1% Triton X-100, 10 mM NEM, protease inhibitors [Roche catalog no. 11697498001]). Insoluble cell debris was removed by centrifuging lysed cells at 16,100 × *g* and discarding the pellet. Protein concentration was quantified with the Bradford assay (Bio-Rad).

### Western blotting.

Protein samples were separated by 12% SDS-PAGE. After electrophoresis, the proteins were transferred to a nitrocellulose membrane (pore size, 0.2 µm; MilliporeSigma) in Towbin transfer buffer (25 mM Tris, 192 mM glycine, 20% methanol) at 60 V overnight. Membranes were blocked with 2% nonfat milk in PBS-T buffer (144 mg/liter KH_2_PO_4_, 9 g/liter NaCl, 795 mg/liter Na_2_HPO_4_ [pH 7.4], 0.1% Tween 20) for 1 h. Membranes were probed with primary and secondary antibodies diluted in 2% milk in PBS-T as follows: anti-TAg antibody (pAb416) at a 1:5,000 dilution (93), anti-syntaxin 18 antibody (Abcam, Inc., ab156017) diluted in 5% bovine serum albumin at 1:1,000, anti-Rab18 antibody (MilliporeSigma SAB4200173) at 1:10,000, anti-VP1 antibody (pAb5G6) at 1:1000, anti-GAPDH antibody (Abcam, Inc., ab9484) at 1: 10,000, anti-ZW10 kinetochore protein antibody (Abcam, Inc., ab53676) at 1:300, anti-RAD50 interactor 1 antibody (MilliporeSigma HPA019875) at 1:200, anti-β-actin antibody (Cell Signaling 4967) at 1:10,000, horseradish peroxidase (HRP)-conjugated ECL sheep anti-mouse (GE Healthcare NA931V) at 1:5,000, and HRP-conjugated ECL donkey anti-rabbit antibody (GE Healthcare NA934V) at 1:5,000. Protein bands were visualized with HRP substrate (Millipore WBLUF0100) and exposure to X-ray film or the Syngene PXi gel doc system.

### Immunofluorescent staining.

Eighteen-millimeter circular coverslips (no. 1.5 thickness; Electron Microscopy Sciences catalog no. 72222) were coated with 0.1% poly-l-lysine in water (MilliporeSigma catalog no. P8920) in 12-well plates for 5 min at room temperature. The coverslips were then rinsed with cell culture grade water and allowed to dry for at least 2 h. RPTE cells were then seeded onto the coverslips in the 12-well plate. For processing, the cells were fixed with 4% paraformaldehyde at room temperature for 20 min. Antigen was retrieved with antigen retrieval buffer (100 mM Tris, 5% [wt/vol] urea [pH 9.5]) at 95°C for 10 min. Cell membranes were permeabilized with 0.1% Triton X-100 in PBS at room temperature for 5 min. The coverslips were blocked with 5% goat serum in PBS for 1 h and probed with diluted primary and secondary antibodies successively for 1 h each. The coverslips were washed three times with PBS between steps. Finally, the coverslips were mounted with Prolong Gold Reagent with 4',6-diamidino-2-phenylindole (DAPI; Thermo Fisher Scientific catalog no. P36931). Primary and secondary antibodies were diluted in 5% goat serum (MilliporeSigma) as follows: anti-VP1 antibody (pAb5G6) at 1:200, anti-Rab18 antibody (MilliporeSigma SAB4200173) at 1:1500, anti-Rab7 antibody (Cell Signaling 9367) at 1:100, anti-GM130 antibody (Cell Signaling 12480) at 1:200, anti-EEA1 antibody (Cell Signaling 3288) at 1:200, anti-Golgin-97 antibody (Cell Signaling 13192) at 1:100, goat anti-mouse IgG-FITC antibody (MilliporeSigma F2012) at 1:200, and goat anti-rabbit IgG-DL594 antibody (Thermo Fisher Scientific 35561) at 1:200. Images were taken with an Olympus BX41 or a Leica inverted SP5 confocal microscope system with a 100× objective. Confocal images were acquired and processed with Leica Application Suite (LAS) AF and LAS X software.
