# Supplementary material for: Identification of Rab18 as an Essential Host Factor for BK Polyomavirus Infection Using a Whole-Genome RNA Interference Screen
Source: mSphere. 2017 Jul 26;2(4):e00291-17. doi: 10.1128/mSphereDirect.00291-17 (PMC5555678; doi:10.1128/mSphereDirect.00291-17)
Supplement: TABLE S2 [file sph004172331st2.docx]

| Term | P Values | Fold Enrichment |
| --- | --- | --- |
| Translational elongation | 2.68E-13 | 6.93517778 |
| Translation | 2.07E-08 | 3.060194567 |
| Vesicle coating | 8.47E-06 | 12.81081451 |
| Membrane budding | 1.31E-05 | 12.0101386 |
| Golgi transport vesicle coating | 1.35E-05 | 16.47104723 |
| COPI coating of Golgi vesicle | 1.35E-05 | 16.47104723 |
| Golgi vesicle budding | 1.35E-05 | 16.47104723 |
| Vesicle targeting, to, from or within Golgi | 3.99E-05 | 13.72587269 |
| Vesicle targeting | 1.02E-04 | 8.734646257 |
| Vesicle organization | 4.63E-04 | 4.844425655 |
| Retrograde vesicle-mediated transport, Golgi to ER | 7.80E-04 | 7.843355823 |
| Establishment of vesicle localization | 0.001073955 | 5.823097505 |
| Intracellular transport | 0.001376634 | 1.694552184 |
| Cellular protein localization | 0.001761447 | 1.902596214 |
| Cellular macromolecule localization | 0.001968891 | 1.888572164 |
| Vesicle localization | 0.001993902 | 5.19357345 |
| Intracellular protein transport | 0.002056658 | 1.939525489 |
